# An exploration of the Facebook social networks of smokers and non-smokers

**DOI:** 10.1371/journal.pone.0187332

**Published:** 2017-11-02

**Authors:** Luella Fu, Megan A. Jacobs, Jody Brookover, Thomas W. Valente, Nathan K. Cobb, Amanda L. Graham

**Affiliations:** 1 Marshall School of Business, University of Southern California, Los Angeles, California, United States of America; 2 Schroeder Institute for Tobacco Research and Policy Studies at Truth Initiative, Washington, DC, United States of America; 3 Department of Preventive Medicine, Keck School of Medicine, University of Southern California, Los Angeles, California, United States of America; 4 Department of Pulmonary and Critical Care, Georgetown University Medical Center, Washington, DC, United States of America; 5 Department of Oncology, Georgetown University Medical Center/Cancer Prevention and Control Program, Lombardi Comprehensive Cancer Center, Washington, DC, United States of America; Universite Toulouse 1 Capitole, FRANCE

## Abstract

**Background:**

Social networks influence health behavior, including tobacco use and cessation. To date, little is known about whether and how the networks of online smokers and non-smokers may differ, or the potential implications of such differences with regards to intervention efforts. Understanding how social networks vary by smoking status could inform public health efforts to accelerate cessation or slow the adoption of tobacco use.

**Objectives:**

These secondary analyses explore the structure of ego networks of both smokers and non-smokers collected as part of a randomized control trial conducted within Facebook.

**Methods:**

During the trial, a total of 14,010 individuals installed a Facebook smoking cessation app: 9,042 smokers who were randomized in the trial, an additional 2,881 smokers who did not meet full eligibility criteria, and 2,087 non-smokers. The ego network for all individuals was constructed out to second-degree connections. Four kinds of networks were constructed: friendship, family, photo, and group networks. From these networks we measured edges, isolates, density, mean betweenness, transitivity, and mean closeness. We also measured diameter, clustering, and modularity without ego and isolates. Logistic regressions were performed with smoking status as the response and network metrics as the primary independent variables and demographics and Facebook utilization metrics as covariates.

**Results:**

The four networks had different characteristics, indicated by different multicollinearity issues and by logistic regression output. Among Friendship networks, the odds of smoking were higher in networks with lower betweenness (p = 0.00), lower transitivity (p = 0.00), and larger diameter (p = 0.00). Among Family networks, the odds of smoking were higher in networks with more vertices (p = .01), less transitivity (p = .04), and fewer isolates (p = .01). Among Photo networks, none of the network metrics were predictive of smoking status. Among Group networks, the odds of smoking were higher when diameter was smaller (p = .04). Together, these findings suggested that compared to non-smokers, smokers in this sample had less connected, more dispersed Facebook Friendship networks; larger but more fractured Family networks with fewer isolates; more compact Group networks; and Photo networks that were similar in network structure to those of non-smokers.

**Conclusions:**

This study illustrates the importance of examining structural differences in online social networks as a critical component for network-based interventions and lays the foundation for future research that examines the ways that social networks differ based on individual health behavior. Interventions that seek to target the behavior of individuals in the context of their social environment would be well served to understand social network structures of participants.

## Introduction

It is well established that social networks influence health behavior, and that an individual’s health behavior can impact the network itself [1–3]. These relationships are particularly well documented for tobacco use, where social network structure has been associated with smoking initiation, continuation, and cessation [4, 5]. Adolescents who are peripheral or isolated within social networks are more likely to smoke, and peripheral network position often precedes the adoption of smoking [6]. Tobacco use may contribute to the maintenance of network ties among friends and family, while its discontinuation may cause ties to fray or fracture [7–10]. Following smoking cessation, it may be that new ties are formed resulting in larger nonsmoking networks [11]. In adults, smoking cessation has been shown to spread over time through social networks [12]. This dynamic interplay between social networks and health behavior has formed the basis for many network-based health promotion and tobacco control efforts [1, 13].

Historically, an inherent challenge in network-based behavior change interventions involved enumerating the network itself. Individuals’ personal networks, or ego networks, are comprised of not only whom an individual knows, but also the ties between and among those other individuals. A single individual may be able to list his/her contacts, but may be uncertain as to which friends know one another. Asking an individual to identify all potential pairs of ties within their network can be both exhausting (a network with 100 friends has 4,950 potential ties) and also inaccurate, since this approach assumes the individual is aware of all potential ties. As a result, studies of health behavior in social networks often involve incomplete networks [12].

Online social networks provide an alternative setting for exploring more complete ego-level networks. Networks such as Facebook can provide a complete inventory of “friends” and the ties between friends, enabling an unprecedented and automated view of egocentric networks at scale. In Facebook and other online social networks, ties between friends are drawn from the master network and represent nominations by those friends rather than assumptions on the part of the individual at the center of the network. Online networks also allow for the exploration of the role of specific types of network ties in influencing health behavior. Users can designate network ties as being members of a specific type of network, such as family members. Whether through genetics, behavioral observation, or rules about smoking in the home environment, family networks are known to exert strong influences on tobacco use behavior [14, 15]. To the extent that online networks often encompass myriad types of social ties, they may offer a richer or more accurate depiction of an individual’s social network through alternative mechanisms for tie-detection or weighting such as co-occurrences in photographs or memberships in affinity groups. These systems thus represent a parallel representation of an individual’s network with unique advantages, including its near-real time availability to support interventions [16].

Indeed, online social networks are increasingly being employed for tobacco marketing [17, 18], tobacco control [19–21] and smoking cessation interventions [22–27]. These efforts typically leverage the scalability and accessibility of online social networks to reach and engage tobacco users, but often provide few insights into the nature of the online networks themselves. A noteworthy exception is a recent study by Cole-Lewis et al. [28] conducted within the Smokefree Women Facebook group. The authors visualized the Facebook network both with and without the moderator, demonstrating the important role of the moderator and also of a small group of “super participants” in connecting with less engaged group members. Centrality of group members was correlated with self-reported abstinence. As noted by Latkin and Knowlton [13], “it can be important to understand the structure and stability of social networks before and during social diffusion interventions” (page 10). To date, little is known about whether and how the networks of online smokers and non-smokers may differ, or the potential implications of such differences with regards to intervention efforts. Understanding how social networks vary by smoking status could inform public health efforts to accelerate cessation or slow the adoption of tobacco use.

To begin to address this gap, this study sought to leverage the availability of a unique social network dataset of complete egocentric networks of adult smokers and non-smokers collected as part of a randomized control trial conducted within Facebook. Specifically, we were interested in documenting the structural characteristics of the online social networks of smokers and non-smokers and exploring the relationship of structural differences to smoking status. These secondary analyses from the trial are largely exploratory since there is very little empirical work or even theory to guide us. Prior research comparing the networks of smokers and non-smokers has exclusively focused on the smoking behavior of those networks, and has not addressed broad structural comparisons. These studies have also mostly been conducted in schools, among adolescents, and (offline) focused friendships. Although smokers and non-smokers differ on some sociodemographic characteristics, it is not known whether their social networks differ. While datasets of inter-related complete egocentric networks are common in industry (e.g., Facebook, online games, other programs on online social networks), we are aware of no health-related research that has accessed and analyzed these networks. The aim of these analyses was to explore whether there are network level differences between smokers and non-smokers that might have implications for the design and delivery of public health campaigns and social network-based cessation interventions.

## Methods

### Recruitment and enrollment

The trial [29, 30] was conducted entirely within Facebook between December 2012 and October 2013. The study protocol was approved by Schulman Associates institutional review board (formerly Independent IRB). All participants were registered users of Facebook and were recruited via Facebook advertising and earned media. Individuals that clicked through to the Facebook smoking cessation application (“UbiQUITous”) tested in the trial were shown a dialog box asking for installation permission, followed by an informed consent screen for study participation. Inclusion criteria for the trial were US residency, current smoking, age 18 or older, an active English-language Facebook account and an email address, acceptance of Facebook permissions for application install, and provision of informed consent. Age, number of existing friends that were already application users, and location-related eligibility were retrieved from Facebook at app installation; smoking status was determined via self-report immediately after informed consent. Individuals who met eligibility criteria and were randomized were considered “study seeds” in the trial.

Study seeds were randomized to one of 12 variants of the app in a fractional factorial design. Full details about each cell of the factorial have been previously described [29, 30]. Of particular relevance to these analyses were the cells in the factorial that enabled study seeds to invite nonsmokers to participate in the app to help support their efforts in quitting smoking.

Tracking tags were embedded within all links to the application and in content shared by users, enabling per-user tracking of diffusion. New users that reached the application through an existing study seed were identified in real-time and excluded from becoming seeds themselves. Descendants were users–both smokers and non-smokers–that could be tied to an existing participant through a tracking mechanism. Descendants installed the application and accepted informed consent in the same manner as seed users. Descendants who did not have a verified source were attributed as descendants of the friend who had most recently installed the app (“guessed parent”).

Seed users accepted app permissions in addition to providing informed consent to participate in the study. The UbiQUITous app permissions included standard Facebook permissions to pull social network data from friends and friends-of-friends. Participants’ friends and friends-of-friends who had their individual Facebook settings configured to enable this type of data sharing were thus included in the dataset.

During the trial, a total of 14,010 individuals installed the UbiQUITous app, accepted informed consent, and indicated their smoking status. These individuals comprise the analytic sample for the present analyses. Included in the analytic sample are the 9,042 smokers (“study seeds”) who went on to be randomized in the trial [29], an additional 2,881 smokers who did not meet full eligibility criteria (e.g., missing Facebook data, incomplete baseline assessment, non-U.S. residence, under age 18) and were not randomized, and 2,087 non-smokers. All individuals had full access to the Facebook application.

### Data collection and measures

Data collection occurred primarily through Facebook’s application programming interface (API). The API allowed our systems to interact directly with Facebook’s database to retrieve data about individual users (e.g., age, gender) and their immediate social network upon study enrollment. At app install, users accepted Facebook’s app install permissions, which included access to their profile (e.g., birthday, gender, hometown, relationship details), photo information (e.g., number of photos tagged, names of friends tagged within photos), group membership information (e.g., name of Group, membership of others in Group), list of friends and friends’ profile information (e.g., birthday, relationship details, photos). The system automatically retrieved each participant’s complete ego network and stored the data in a relational database, including a list of their friends and the ties between those friends (i.e., second-degree connections). We also extracted three metrics of Facebook utilization: 1) count of “likes” (number of Facebook pages an individual liked), 2) wall post count (number of posts an individual wrote to their own wall), and 3) page views (total count of pages viewed in app). Likes and wall posts were extracted at app installation, and page views was extracted 30 days after app installation.

### Analytic plan

For each individual in the analytic sample, we constructed a complete egocentric network by integrating ties between a participant’s friends as provided by Facebook. Eleven network metrics were used in this study: vertices, edges, density, isolates, diameter, communities, betweenness centrality, closeness centrality, transitivity, clusters, and modularity. The first four metrics are descriptive metrics of how large a network is and how many connections it contains. These are lower order network structural terms that are a necessary first step in characterizing a network. The remaining seven metrics are higher order structural measures that can be classified into three types: 1) cohesion (diameter, communities), or how compact a networks is; 2) clustering (transitivity, clusters, and modularity), or the extent to which a network is characterized by having distinct, separate pockets of dense interconnectivity separated by bridges; and 3) centrality (closeness, betweenness), or whether there are some individuals that are particularly prominent or central in the network. We calculated these eleven social network metrics using the using the iGraph 1.0.1 package for R (Table 1) [31].

**Table 1:** Social network metrics of interest and their definition.

Metric	Definition
Vertices	Number of Facebook friends of an ego (i.e., network size); an ego-level metric
Edges	Number of friendships in the network, including friendships between an ego’s friends; a network-level metric
Density*	The portion of potential connections in a network that are actual connections (calculated as the existing friendships over all possible friendships); a network-level metric
Isolates	Number of individuals with no friends other than the ego; a network-level metric
Diameter*	Maximum degree of separation between any two individuals in the network; a network-level metric
Communities*	Number of groups, sorted to increase dense connections within the group and decrease sparse connections outside it (i.e., to maximize modularity); a network-level metric
Closeness*	Average of how closely associated members are to one another; a network-level metric
Betweenness*	An average of the relative importance of all individuals within their own network, normalized as 2*betweenness/(vertices-1)(vertices-2); a network-level metric
Transitivity*	The extent to which the relationship between two nodes in a network that are connected by an edge is transitive (calculated as the number of triads divided by all possible connections); a network-level metric
Clusters*	Number of subnetworks; a network-level metric
Modularity	The strength of division of a network into communities (calculated as the fraction of ties between community members in excess of the expected number of ties within communities if ties were random); a network-level metric

* calculated after removing ego and isolates

We constructed these 11 metrics for four Facebook networks: Friendship, Family, Photo Association, and Group Association networks. Since Facebook allows users to identify friends as family members, we could label ties as being familial to enable the construction of Family networks. The Photo Association network for each individual was constructed using tags in participants’ Facebook photographs. The Group Association network was constructed using information about Facebook groups that participants joined and the membership of those groups.

Next, we examined the multicollinearity among the 11 network metrics to determine which variables to examine as predictors of smoking status. Multicollinearity was assessed using the variance inflation factor using the vif function in the R package [32]. The vif is a generalization of correlation: while correlation only shows the relationship between pairs of variables, vif indicates how related one independent variable is to all the other independent variables. If a variable had a vif value above 10, we removed some of the correlated variables based on information from the correlation matrix. We considered a correlation at or above 0.8 as high. In determining which one of a pair of highly correlated variables to remove from regression analyses, we chose the variable that was also highly correlated with other variables. This approach allowed us to reduce multicollinearity while retaining potentially informative variables.

Using a streamlined set of predictors, we conducted a series of logistic regressions using R software (v. 3.2.2) to analyze the relationship of network metrics and smoking status in each of the four Facebook networks. To control for variables that might confound the association of network metrics and smoking status, we included gender (female, male, or unknown), age, country (US, Canada, Mexico, or other), and two metrics of intensity of Facebook utilization (“likes”, wall posts) and page views as a metric of app utilization. All independent variables were rescaled to mean of zero and standard deviation of one, and the significance level was .05.

## Results

### Sample characteristics

Compared to non-smokers, smokers were older (median [IQR]: 46 [21] vs 35 [27], p = 0.00), more likely to be female (70% vs. 63%, p = 0.00), and more active Facebook users based on friends (208 [312] vs. 152 [274], p = 0.00), “likes” (237 [475] vs 120 [407], p = 0.00), and wall posts (109 [194] vs 52 [148], p = 0.00).

### Multicollinearity among social network metrics by Facebook network type

Table 2 shows the correlation matrix among the 11 social network metrics within each of the four Facebook networks. Demographic variables (age, gender) and Facebook wall posts were included in the original correlation matrix, but their correlations with each other and the network metrics were consistently below 10% and therefore were omitted. Multicollinearity varied across the four types of Facebook networks, showing that the underlying social relationships within each network give rise to different network structures. Correlations that were significant at greater than 0.8 were observed for the following variables. In Friendship networks, closeness (Cn) and density (Dn) were correlated at .98; community (Cm) and clustering (Ct) were correlated at .91. In Family networks, vertices (V) was correlated with edges (E) at .92 and with community (Cm) at .80; closeness (Cn) was correlated with density (Dn) at .99 and with transitivity (T) at .90. A similar pattern emerged in Photo Association networks: vertices (V) was correlated with edges (E) at .89; closeness (Cn) was correlated with density (Dn) at .99 and with transitivity (T) at .92. In Group Association networks, density (Dn) was associated with closeness (Cn) at .99 and with modularity (M) at -.81.

**Table 2:** Correlation matrices for Facebook friendship, family, Photo Association, and Group Association network metrics.

**Friendship Networks**											
	V	E	I	Dm	Dn	T	Ct	B	Cn	Cm	M
V	1.00										
E	.59	1.00									
I	.70	.06	1.00								
Dm	.39	.15	.10	1.00							
Dn	-.33	-.07	-.15	-.56	1.00						
T	-.22	.08	-.24	-.37	.74	1.00					
Ct	.48	.11	.41	.53	-.36	-.38	1.00				
B	-.36	.-19	-.1	-.46	.55	.19	-.23	1.00			
Cn	-.29	-.08	-.12	-.51	**.98**	.67	-.30	.51	1.00		
Cm	.56	.19	.33	.67	-.47	-.45	**.91**	-.34	-.40	1.00	
M	-.01	-.20	.04	.39	-.56	-.62	.29	-.14	-.51	.31	1.00
**Family Networks**											
V	1.00										
E	**.92**	1.00									
I	.63	.34	1.00								
Dm	.71	.67	.21	1.00							
Dn	-.67	-.48	-.68	-.49	1.00						
T	-.46	-.23	-.68	-.27	.92	1.00					
Ct	.44	-.26	.34	.15	-.42	.15	1.00				
B	-.41	-.44	-.04	-.4	-.12	-.37	-.16	1.00			
Cn	-.63	-.46	-.61	-.49	**.99**	**.90**	-.39	-.21	1.00		
Cm	**.80**	.73	.32	.74	-.57	.74	.57	-.43	-.54	1.00	
M	.59	.45	.30	.52	-.59	.52	.76	-.27	-.56	.72	1.00
**Photo Association Networks**											
V	1.00										
E	**.89**	1.00									
I	.87	.59	1.00								
Dm	.53	.65	.18	1.00							
Dn	-.70	-.54	-.67	-.36	1.00						
T	-.64	-.32	-.64	-.16	.94	1.00					
Ct	.53	.44	-.6	.23	-.4	-.31	1.00				
B	-.54	-.54	-.39	-.41	.11	-.08	-.3	1.00			
Cn	-.64	-.51	-.60	-.36	**.99**	**.92**	-.37	.002	1.00		
Cm	.68	.75	.33	.72	-.46	-.26	.70	-.47	.33	1.00	
M	.55	.50	.33	.50	-.48	-.36	.82	-.37	.33	.75	1.00
**Group Association Networks**											
V	1.00										
E	.86	1.00									
I	.03	-.02	1.00								
Dm	.56	.30	.09	1.00							
Dn	-.34	-.08	-.41	-.60	1.00						
T	.00	.07	-.41	-.28	.73	1.00					
Ct	.16	.01	.17	.12	-.57	-.27	1.00				
B	-.33	-.22	.32	-.19	-.31	-.71	.06	1.00			
Cn	-.35	-.09	-.39	-.59	**.99**	.71	-.54	-.33	1.00		
Cm	.52	.26	.14	.65	-.74	-.36	.69	-.12	-.72	1.00	
M	.19	-.01	.12	.43	**-.81**	-.56	.57	.14	-.79	.59	1.00

**Bolded terms** have magnitude greater than .8, indicating multicollinearity.

V = vertices, E = edges, I = isolates, Dm = diameter, Dn = density, T = transitivity, Ct = clusters, B = betweenness, Cn = closeness, Cm = communities, M = modularity.

**Fig 1:**
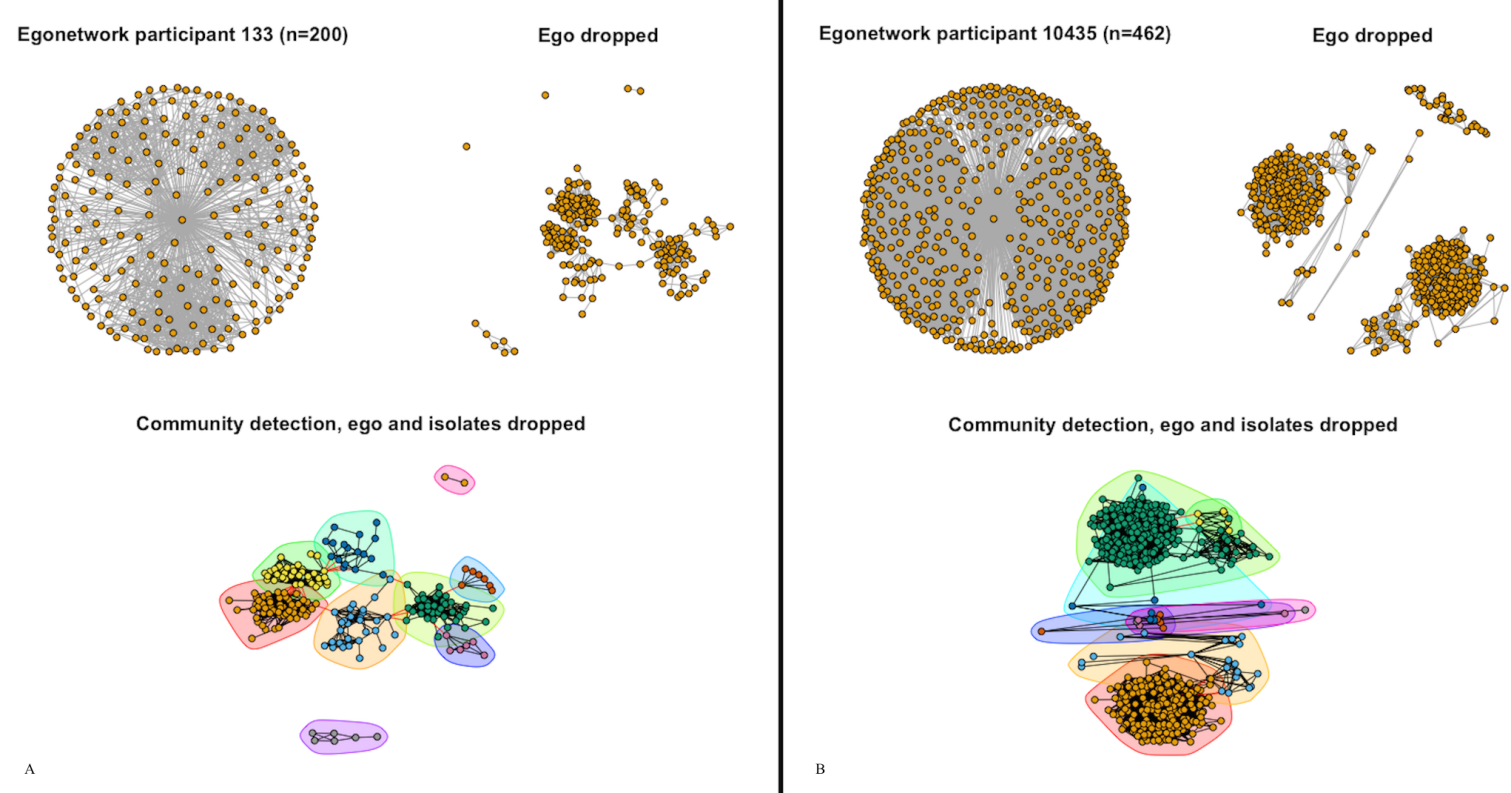
Friendship networks. (1A) A smoker’s friendship network and (1B) a nonsmoker’s friendship network are shown with ego, without ego, and with communities drawn after excluding ego and isolates.

**Fig 2:**
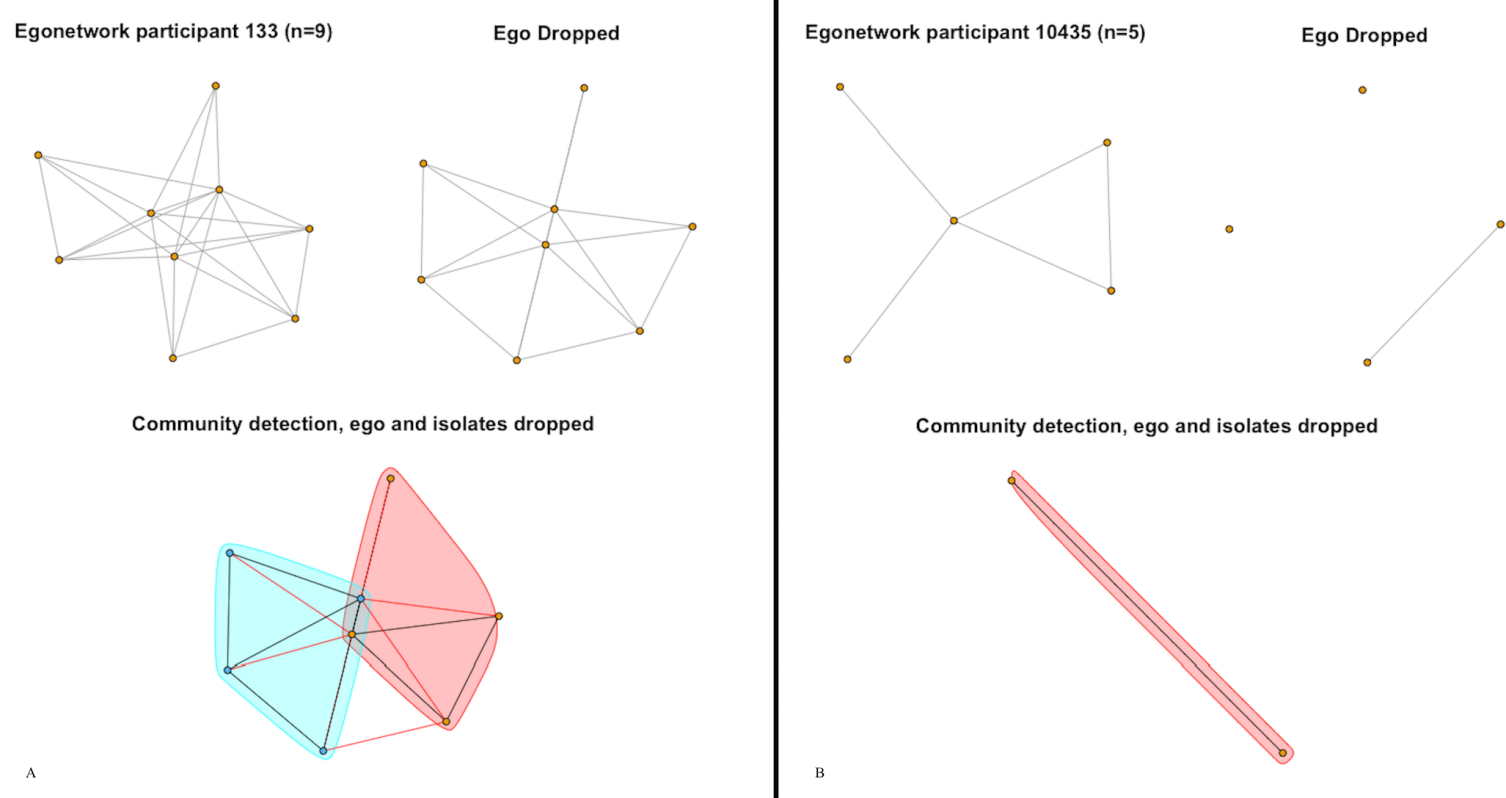
Family networks. (2A) A smoker’s Family network and (2B) a nonsmoker’s Family network are shown with ego, without ego, and with communities drawn after excluding ego and isolates.

Variations in network structures for Friendship, Family, Photo Association, and Group Association networks may help explain differences in their correlation matrices. Friendship networks can be comprised of hundreds of people as shown in both Fig 1A for smokers and Fig 1B for non-smokers. Because Facebook Friendship networks have so many members and vary greatly in how individuals choose to interact, most metrics are not correlated. One particularly high correlation to note, however, is between clusters and communities (91%), which is not present in other networks. Seen in Fig 1, clusters play an important part in differentiating individuals’ friendship networks; it makes sense that clusters would form for different communities of friends. In contrast, a weaker connection between clusters and communities (57%) is reasonable for a family network, where it is more likely that all family members form one community as in Fig 2B. Together, these metrics suggest that Friendship networks tend to be large, have various friendship patterns, and cluster in ways that greatly influence communities.

**Fig 3:**
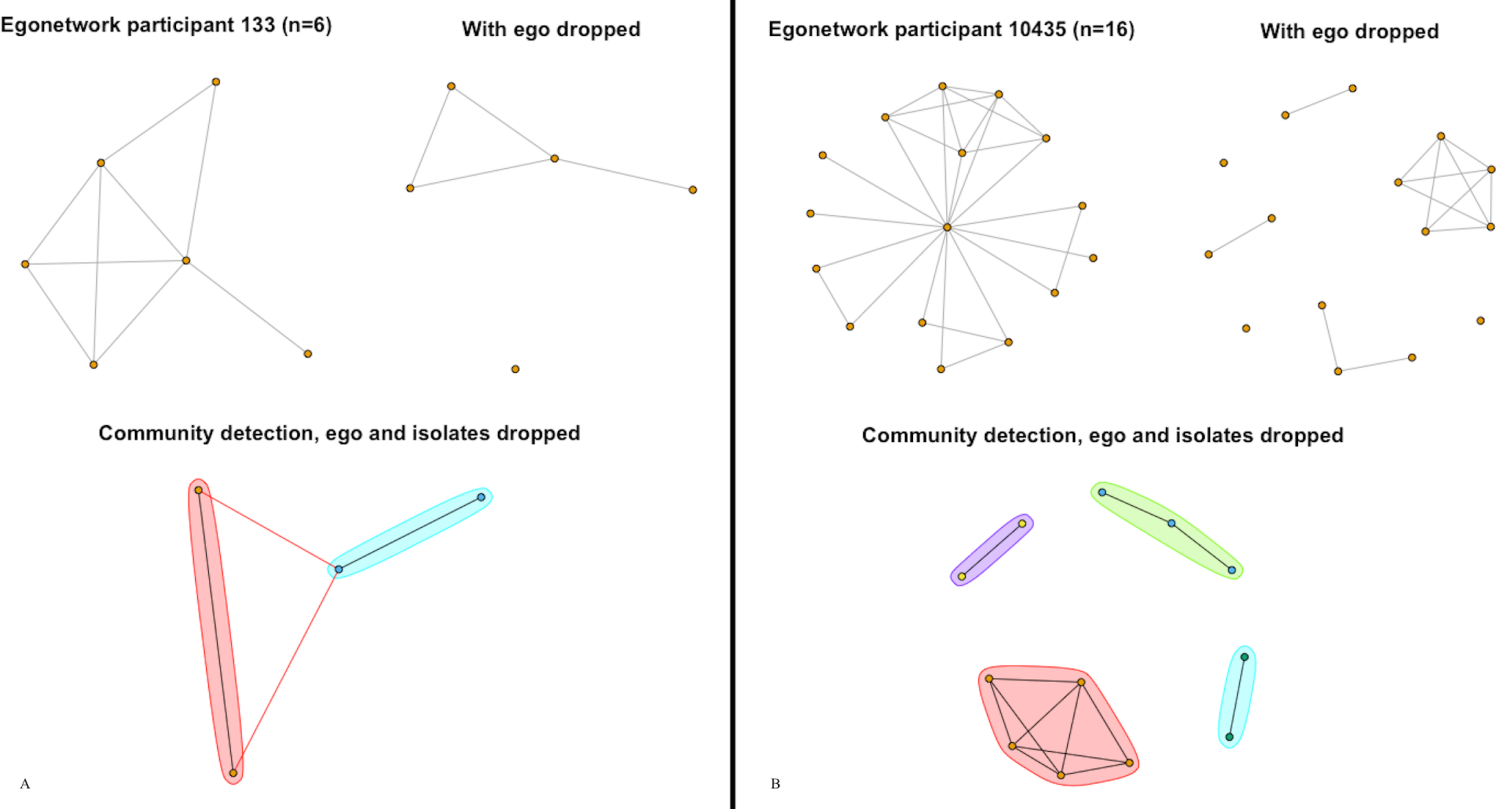
Photo association networks. (3A) A smoker’s photo network and (3B) a nonsmoker’s photo network are shown with ego, without ego, and with communities drawn after excluding ego and isolates.

Family networks tend to be small, with greater transitivity signaling family members that are closer to each other. The Family networks shown in both Fig 2A and 2B both have fewer than 10 members. This size may explain the high correlation between transitivity and closeness (.90). In small networks like these, triads greatly impact network closeness. Comparing Fig 2A (a Family network among smokers where transitivity is higher) to Fig 2B (a Family network among non-smokers where transitivity is lower), it is apparent that family members in Fig 2A are also closer on average. A similar pattern is observed among Photo Association networks (Fig 3), where network size is small (between 0–20 members) and transitivity and closeness are highly correlated (.92). Like Family networks, Photo Association networks are usually small and triads affect closeness by bringing the photo members connected to the ego closer to each other.

**Fig 4:**
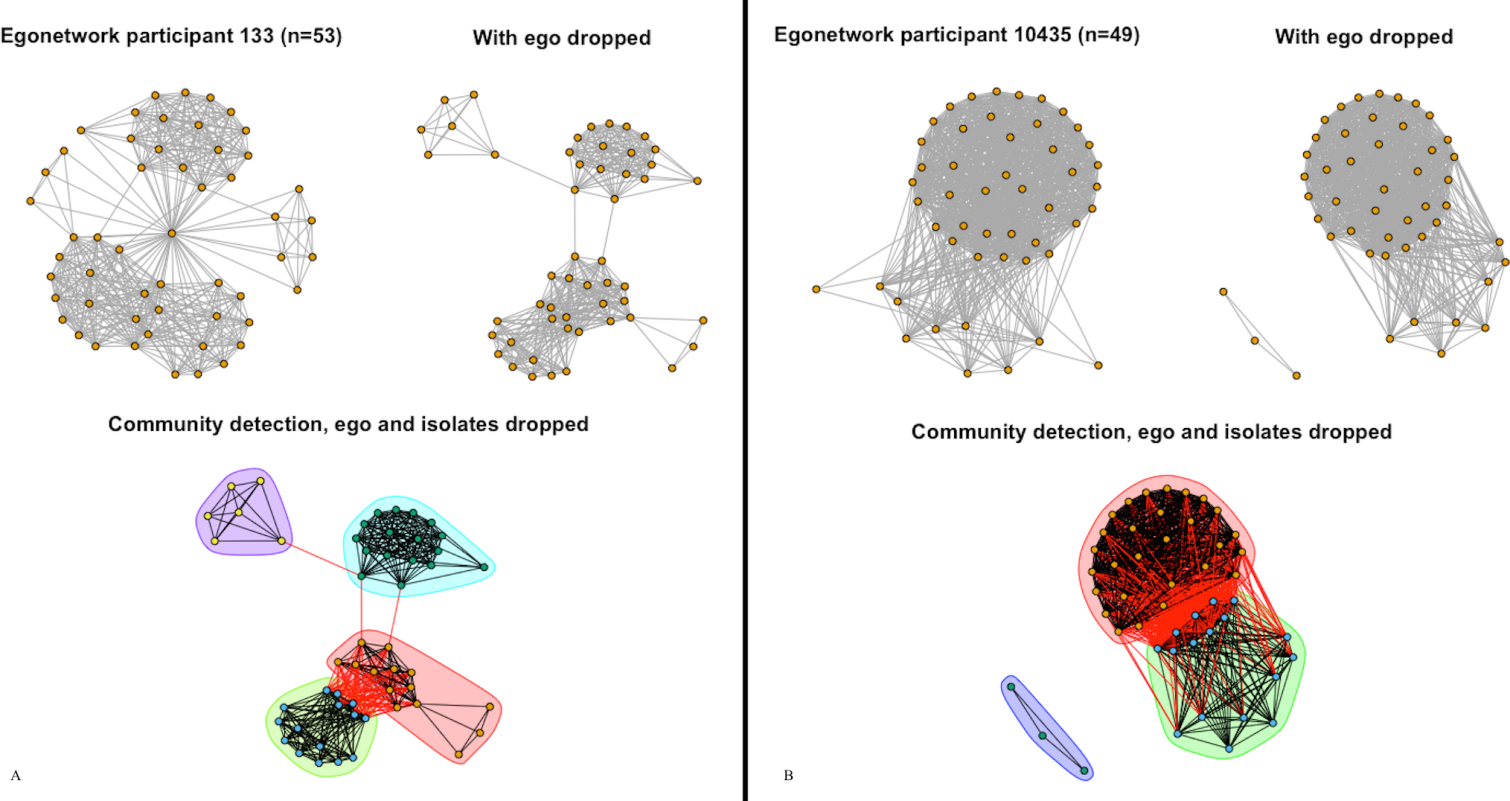
Group Association networks. (4A) A smoker’s group network and (4B) a nonsmoker’s group network are shown with ego, without ego, and with communities drawn after excluding ego and isolates.

In Group Association networks, a distinctive feature is the strong negative correlation between modularity and density (-.81) not seen in other networks. Recall that an edge in this network indicates that two people joined the same group; it is natural that Facebook groups would form the communities being detected. Yet, because people can join multiple groups, the more overlap in membership there is across groups, the more difficult it becomes to differentiate these communities. Higher density may indicate more overlapping membership, thereby lowering modularity. Fig 4 illustrates two networks where the density is higher in Fig 4B (nonsmoking network) than in Fig 4A (smoking network), which also makes the communities harder to see in Fig 4B than in Fig 4A when the colors are ignored.

Based on high vif values and correlations, the following variables were removed from logistic regression analyses. For Friendship networks, density and communities were removed. For Family networks, edges, density, transitivity, closeness, isolates, and clusters were removed. For Photo Association networks, edges, transitivity, closeness, isolates, clusters were removed. In Photo Association networks, vertices were highly correlated with other metrics but were retained in the model because it fundamentally describes the network; instead, we removed the variables that were highly correlated with vertices. For Group Association networks, edges and density were removed.

### Association of social network metrics and smoking status within Facebook networks

Table 3 shows the results of logistic regression analyses for each of the four Facebook networks. In Friendship networks, the odds of smoking increased by 1.13 (1.04, 1.22) for each unit increase in diameter, whereas the odds of smoking decreased by 0.90 (0.85, 0.95) for each unit increase in betweenness, and by 0.76 (0.70, 0.82) for each unit increase in transitivity. Together these findings indicate that smokers’ Friendship networks were more loosely connected with fewer ties between an ego’s friends and fewer members bridging ties between others.

**Table 3:** Association of friendship, family, photo association, and group association network metrics with smoking status (Odds Ratio, 95% CI).

	Friendship	Family	Photo Association	Group Association
Predictors				
Vertices	0.88 (0.76, 1.02)	**1.26 (1.07, 1.50)**	0.87 (0.67, 1.17)	1.11 (1.00, 1.25)
Edges	1.03 (0.95, 1.14)	--	--	--
Density	--	--	0.99 (0.79, 1.26)	--
Betweenness	**0.90 (0.85, 0.95)**	--	0.93 (0.77, 1.13)	1.01 (0.86, 1.19)
Transitivity	**0.76 (0.70, 0.82)**	**0.88 (0.78, 1.00)**	--	1.07 (0.89, 1.28)
Closeness	0.98 (0.92, 1.05)	--	--	0.83 (0.66, 1.04)
Isolates	1.03 (0.93, 1.15)	**0.82 (0.71, 0.95)**	--	1.02 (0.93, 1.13)
Diameter	**1.13 (1.04, 1.22)**	--	1.13 (0.90, 1.42)	**0.86 (0.74, 0.99**)
Clusters	1.06 (0.98, 1.15)	--	--	1.03 (0.88, 1.21)
Communities	--	--	1.05 (0.76, 1.47)	1.00 (0.82, 1.23)
Modularity	0.95 (0.90, 1.02)	1.08 (0.96, 1.23)	0.99 (0.78, 1.26)	1.01 (0.86, 1.19)
Covariates				
Age (years)	**1.61 (1.53, 1.70)**	**1.24 (1.13, 1.37)**	**1.36 (1.16, 1.59)**	**1.60 (1.47, 1.74**)
Gender: Unknown	1.05 (0.56, 2.14)	1.02 (0.40, 3.47)	1.65 (0.32, 30.42)	1.77 (0.63, 7.43)
Gender: Male	1.02 (0.92, 1.14)	1.02 (0.83, 1.26)	0.89 (0.64, 1.26)	0.92 (0.78, 1.08)
Country: Canada	**0.37 (0.17, 0.88)**	**0.14 (0.03, 0.62)**	0.10 (0.00, 1.03)	0.38 (0.13, 1.25)
Country: Mexico	**0.36 (0.17, 0.76)**	**0.12 (0.02, 0.75)**	--	0.46 (0.13, 1.82)
Country: Other	0.93 (0.84, 1.03)	0.91 (0.76, 1.08)	1.11 (0.83, 1.49)	0.99 (0.85, 1.15)
Likes Count	0.97 (0.93, 1.03)	0.96 (0.89, 1.04)	**1.24 (1.03, 1.55)**	1.00 (0.93, 1.09)
Wall Count	**1.14 (1.07, 1.23)**	0.98 (0.91, 1.07)	1.15 (0.96, 1.41)	1.08 (0.99, 1.19)
Page View Count	**1.88 (1.66, 2.15)**	**1.55 (1.29, 1.90)**	**2.10 (1.44, 3.32)**	**2.13 (1.75, 2.65**)
Intercept	**7.30 (6.73, 7.92)**	**9.45 (8.24, 10.89)**	**8.58 (6.79, 11.01)**	**8.95 (7.90, 10.17)**


**Bolded terms** are significant at the 95% confidence level

“-- “indicates a variable removed due to high correlation

In Family networks, vertices, transitivity, and isolates differentiated smoker and nonsmoker networks. For each unit increase in vertices, the odds of smoking increased by 1.26 (1.07, 1.50). The odds of smoking decreased by 0.88 (0.78, 0.998) for each unit increase in transitivity, and by 0.82 (0.72, 0.95) for each unit increase in isolates. Together, these findings suggest that compared to non-smoker Family networks, those of smokers contained more family members but fewer ties between those members and fewer members who had no ties to the rest of the family. None of the social network metrics differentiated smokers from non-smokers in the Photo Association network. In Group Association networks, for each unit increase in diameter, the odds of smoking decreased by 0.86 (0.75, 0.99), suggesting that smoker Group Association networks were more compact than those of nonsmokers.

## Discussion

This study leveraged a unique dataset of 14,010 complete egocentric networks derived from Facebook and the smoking status of all egos. Access to complete networks allowed us to calculate social network measures such as density, transitivity and betweenness for different types of Facebook networks, and correlate them with smoking behavior. We found that smokers’ Friendship networks were more dispersed, had fewer ties between friends, and fewer members that served as bridges to other network members. Smokers were more likely to have larger Family networks than non-smokers, but those family members were less likely to be connected to each other on Facebook. The social network structure of Photo Association networks did not differ between smokers and non-smokers, but Group Association networks among smokers were more compact than those of non-smokers. This study adds to a very scant literature [28] linking the structure of an online social network to health behavior.

There is considerable evidence that social networks impact people’s health and emerging studies linking online networks and health behavior. Naturally, both scientists and public health practitioners have been keen to harness these influences to improve public health by designing network-based interventions. To do so effectively, etiological work on how networks are structured and how they may differ among sub-populations is required. Our finding that smokers’ networks are larger suggests that tobacco cessation interventions in the form of information or persuasion messages delivered via Facebook would likely reach smokers sooner than non-smokers. Moreover, the higher clustering of smokers’ networks indicates that such interventions would be less likely to be reinforced by smokers’ Facebook networks given their greater fragmentation.

We also found that smokers tended to be slightly older than non-smokers and were heavier Facebook users by metrics of “likes” and “wall posts”. They tended to have identified more friends within Facebook, thus having larger networks. We can only speculate on why these differences might occur. Evolving norms and stigma in the United States around smoking may make face-to-face socialization more difficult for smokers, leading to increased social media use for the same purpose. That smokers’ Friendship and Family networks are less dense and have lower metrics of clustering could indicate that smokers form more connections with people they know less well, or that they avoid forming online connections with people that they know well offline as a form of avoiding self-disclosure. This is supported by our finding that Photo networks did not differ between smokers and non-smokers, as these networks would be expected to more closely mirror real world connections as opposed to Facebook nominations. Further research will be needed to determine if these differences correlate with nicotine addiction itself, or if smoking is a co-traveler with psychological traits that drive the network structure.

Several limitations to this study should be considered. First, since individuals were recruited in the context of a smoking cessation trial, it is possible that the groups of smokers and/or non-smokers are not generalizable to Facebook users or the population in general. Our dataset does not include detailed demographic information on race and socioeconomic status, making it impossible to determine the generalizability of the sample either to Facebook itself or nationally in the United States. Our sample is largely comparable to Facebook users overall, which are 76% female [33] and have a median of 200 friends [34], but older than Facebook’s largest demographic cohort of 18–29 year olds [35]. Second, given the way we recruited study participants, our networks may not be entirely representative. We recruited smokers in the context of an online cessation program and in the process captured many non-smokers (i.e., those that were ineligible for the trial, and those that were invited as supporters by intervention participants). It is possible that non-smokers that know and are connected to smokers via social media are different from the greater population of non-smokers in general. Third, online social networks may provide network representations that are different from traditional network enumeration or that are incomplete for different reasons. Facebook emphasizes personal connections but does not include individuals that are not Facebook members, do not use the Internet, or choose not to connect with a specific individual within that online network. A final limitation is that it may not be possible to replicate these analyses given that Facebook no longer allows access to the data that we used to generate full ego networks.

## Conclusion

This study is one of the first to examine the structural differences in online social networks of smokers and non-smokers. We found that ego level online social networks differ between smokers and non-smokers on Facebook. These differences point to the diversity of information available through online social media and the potential to consider various types of networks (e.g., Friendship, Family) when studying smoking and smoking cessation. Our study raises a number of important questions that should be addressed in future research: 1) Given structural differences in social networks, how might the influence of specific types of network ties be leveraged for health behavior change interventions? 2) Does intervening in one type of network offset or counter potential influences from other types of network ties? 3) Do these differences in network structure reflect something about the nature of tobacco use behavior and/or the addiction itself, or are they a function of psychological or social structures that determine network structure? Although our study has raised (and not answered) these questions and others, it illustrates the importance of examining structural differences in online social networks as a critical component for network-based interventions.
